# Differences in organ dysfunctions between neonates and older children: a prospective, observational, multicenter study

**DOI:** 10.1186/cc9323

**Published:** 2010-11-09

**Authors:** Nawar Bestati, Stéphane Leteurtre, Alain Duhamel, François Proulx, Bruno Grandbastien, Jacques Lacroix, Francis Leclerc

**Affiliations:** 1 Service de Réanimation Pédiatrique, Univ Lille Nord de France, UDSL, EA2694, CHU Lille, Avenue Eugène Avinée, 59037 Lille, France; 2 Department of Biostatistics, Univ Lille Nord de France, UDSL, EA2694, CERIM, CHU Lille, Avenue Eugène Avinée, 59037 Lille, France; 3 Pediatric Intensive Care Unit, Sainte-Justine Hospital, Chemin de la côte Ste Catherine, Montreal (Quebec) H3T 1C5, Canada; 4 Department of Epidemiology and Public Health, Univ Lille Nord de France, UDSL, EA2694, CHU Lille, Avenue Oscar Lambret, 59037, Lille, France

## Abstract

**Introduction:**

The multiple organ dysfunction syndrome (MODS) is a major cause of death for patients admitted to pediatric intensive care units (PICU). The Pediatric Logistic Organ Dysfunction (PELOD) score has been validated in order to describe and quantify the severity of organ dysfunction (OD). There are several physiological differences between neonates and older children. The objective of the study was to determine whether there are differences in incidence of ODs and mortality rate between full-term neonates (age <28 days) and older children.

**Methods:**

In a prospective, observational study, 1806 patients, admitted to seven PICUs between September 1998 and February 2000 were included. The PELOD score, which includes six organ dysfunctions and 12 variables, was recorded daily. For each variable, the most abnormal value was used to define the daily OD. For each OD, the most abnormal value each day and that during the entire stay were used in calculating the daily PELOD and PELOD scores, respectively. The relationships between OD, daily OD, PELOD, daily PELOD and mortality were compared between the two strata (neonates, older children) based on the discrimination power, logistic and multiple regression analyses.

**Results:**

Of the 1806 enrolled patients 171 (9.5%) were neonates. Incidence of MODS and mortality rate were higher among neonates than in older children (14.6% vs. 5.5%, *P* < 10^-7^; 75.4%, vs. 50.9%, *P* < 10^-4^; respectively). Daily PELOD scores were significantly higher in neonates from day 1 to day 4. Daily cardiovascular, respiratory and renal dysfunction scores from day 1 to day 4 as well as the PELOD score for the entire pediatric intensive care unit stay were also significantly higher in neonates. Neurological, cardiovascular, and hepatic dysfunctions were independent predictors of death among neonates while all ODs significantly contributed to the risk of mortality in older children.

**Conclusions:**

Our data demonstrate that incidence of MODS and mortality rate are higher among neonates compared to older children. Neurological, cardiovascular, and hepatic dysfunctions were the only significant contributors to neonatal mortality. Stratification for neonates versus older children might be useful in clinical trials where MODS is considered as an outcome measure.

## Introduction

Multiple organ dysfunction syndrome (MODS) is a major problem in the pediatric intensive care unit (PICU) [1,2]. Several studies have shown that mortality increased with the number of organ dysfunctions (ODs) in critically ill children. Two MODS scores have been developed to describe and quantify the severity of OD in critically ill children: the pediatric logistic organ dysfunction (PELOD) score and the pediatric multiple organ dysfunction score (P-MODS) [3-5]. The neonatal multiple organ dysfunction (NEOMOD) score provides information on ODs influencing mortality during the first 28 days of life among critically ill premature babies [6].

There are several physiological and immunological characteristics that may differentiate the neonatal population [7-11]. For example, neonates are especially vulnerable to sepsis and nosocomial infections [12], which represent a well-known cause of MODS [13]. Human and animal studies have shown differences in organ response to injury between neonates and adults [14,15]. Recently, Typpo and colleagues [16] described the epidemiology of MODS at day 1 in patients older than 1 month across PICUs in the US. There was differential PICU mortality based on age. Infants had the highest overall PICU mortality compared with other age groups, and their increased mortality was supposed to be linked to the increased incidence of MODS [16]. No similar study has considered neonates as an independent age group in comparison with the rest of the pediatric population. The aim of the present study was to determine whether there are differences in mortality, OD incidence estimated by the PELOD score, and OD contribution to mortality, between neonates on one side and older children on the other side.

## Materials and methods

### Population

We included all consecutive patients admitted to seven multidisciplinary tertiary-care PICUs of university-affiliated hospitals (two French, three Canadian, and two Swiss) between September 1998 and February 2000. Exclusion criteria were the following: age of 18 years or older, prematurity, pregnancy, PICU length of stay of less than 4 hours, admission in a state of continuous cardiopulmonary resuscitation without achieving stable vital signs for at least 2 hours, transfer to another PICU, and admission for scheduled procedures normally cared for in other hospital locations. Children with a history of prematurity were not excluded. Ethics committees of all participating hospitals approved the study. The requirement for consent was waived since the study was strictly observational.

### Procedures

Data on PELOD score, which considers six ODs (cardiovascular, respiratory, hematological, neurological, hepatic, and renal dysfunction) and 12 variables, were recorded daily. For each variable, the most abnormal value was used to define the daily organ dysfunction (dOD). For each OD, the most abnormal value each day and that during the entire stay were used in calculating the daily PELOD (dPELOD) and PELOD scores, respectively. The distribution of the day 1 PELOD according to the outcome was analyzed by logistic regression and identified three classes of d_1_PELOD score: low (fewer than 10 points), medium (10 to 19 points), and high (at least 20 points).

As the mortality rate decreased after 7 days, daily analyses were limited to data collected during the first week. Thus, only patients who stayed at least 7 days were included in the analysis of the dPELOD score on day 7, those who stayed at least 6 days were included in the analysis of the dPELOD score on day 6, and so on. The dependent variable was survival at PICU discharge.

### Statistical analyses

Two distinct strata were considered: neonates (age of fewer than 28 days post-term) and older children. The descriptive analyses and comparisons of OD, dOD, PELOD, and dPELOD scores between the two strata were performed. Comparisons were performed with Mann-Whitney test (continuous variables) and chi-square or Fisher exact tests (categorical variables). Kaplan-Meier analysis at PICU discharge and log-rank test were used to compare the survival curves between neonates and older children. The relationships between OD, dOD, PELOD, dPELOD, and mortality were compared between the two strata on the basis of the discrimination power as well as logistic and multiple regression analyses.

Based on the expected probability of death, discrimination describes the power of models to distinguish patients who died from those who survived. To estimate the discrimination of the PELOD and dPELOD scores, we used a receiver operator characteristic curve for each strata and calculated the area under the receiver operator characteristic curve (AUC). It is generally accepted that an AUC of at least 0.7 is acceptable, at least 0.8 is good, and at least 0.9 is excellent [17]. Comparison of AUCs was performed when appropriate [18].

To evaluate the relative weight of the ODs within each strata, a logistic regression model was developed. Independent variables were ordinal variables of each OD, and the dependent variable was PICU mortality. Second, a stepwise multiple regression analysis was developed for each strata, using the PELOD score as the dependent variable. All statistical analyses were done with SAS software (SAS Institute, Inc., Cary, NC, USA). *P *values of less than 0.05 were considered significant.

## Results

There were 2,021 consecutive admissions between September 1998 and February 2000. We excluded 215 patients for the following reasons: admission to the PICU for scheduled procedures normally cared for in another hospital location (117), prematurity (55), PICU length of stay of less than 4 hours (13), incomplete records (13), age (12), still cared for in the PICU at the end of the study (2), palliative care (2), and transfer to another PICU (1). Therefore, we enrolled 1,806 patients, including 171 (9.5%) neonates and 1,635 (90.5%) older children (525 infants: 1 month to less than 1 year; 853 children: 1 to less than 12 years; 257 adolescents: at least 12 years). Neonates were more frequently ventilated and had a higher PRISM (pediatric risk of mortality) score than older children. Organ systems of primary dysfunction on admission were different between the two strata. The population characteristics are presented in Table 1. Neurological primary dysfunction was more frequent in older children compared with neonates, whereas cardiovascular and gastrointestinal primary dysfunctions were more frequent in neonates compared with older children. Trauma, cancer, and allergic/immunologic diseases were more frequent in older children, whereas congenital diseases were more frequent in neonates (Table 1).

**Table 1:** Description of the study patients

Characteristics	Number (percentage)			
	**Neonates**		**Older children**	

Male-to-female ratio	1.16		1.22	
Surgical patients	81	(47.4)	801	(49.0)
Ventilated patients^a^	115	(67)	806	(49)
PRISM score, median (Q1-Q3)^a^	10	(5-16)	5	(2-10)
Administrative length of stay in PICU in days, mean; median (Q1-Q3)^a^	8.0; 6	(3-6)	5.5; 3	(2-6)
Organ system of primary dysfunction on admission^b^				
Respiratory	63	(36.8)	568	(34,7)
Neurologic	16	(9.4)	319	(19.5)
Cardiovascular	60	(35.1)	425	(26.0)
Hepatic	1	(0.6)	33	(2.0)
Genitourinary	4	(2.3)	31	(1.9)
Gastrointestinal	20	(11.7)	71	(4.3)
Endocrine	1	(0.6)	21	(1.3)
Musculoskeletal	0	(0.0)	68	(4.2)
Hematologic	1	(0.6)	23	(1.4)
Miscellaneous/undetermined	5	(2.9)	76	(4.7)
Primary category of illness on admission				
Infection	34	(19.9)	405	(24.8)
Trauma^a^	1	(0.6)	174	(10.6)
Congenital disease^a^	102	(59.7)	561	(34.3)
Chemical injury	0	(0.0)	25	(1.5)
Drug	1	(0,6)	11	(0,7)
Cancer^b^	1	(0.6)	59	(3.6)
Diabetes	0	(0.0)	18	(1.1)
Allergic/Immunologic diseases^b^	0	(0.0)	42	(2.6)
Miscellaneous/Undetermined	32	(18.7)	340	(20.8)
Deaths^a^	25	(14.6)	90	(5.5)

^a^*P *< 10^−4^; ^b^*P *< 0.05. PICU, pediatric intensive care unit; PRISM, pediatric risk of mortality; Q1-Q3, first and third quartile.

The global case-fatality rate was 6.4% (115 deaths). Mortality was higher among neonates compared with older patients (14.6% versus 5.5%, *P *< 10^−7^). Given different age subgroups of older children (infant: 1 month to 1 year), toddler and preschool (2 to 5 years), school-age child (6 to 12 years), and adolescent and young adult (13 to 18 years), mortality rate was not different (6.6%, 4.6%, 4.8%, 5.8%, respectively; *P *= 0.48). Kaplan-Meier analysis at PICU discharge showed that the mortality rate was significantly higher in neonates compared with older children (log-rank *P *= 0.003). MODS was also significantly more frequent in neonates compared with older children (75.4% versus 50.9%, *P *< 10^−4^).

### PELOD and daily PELOD scores

**Figure 1:**
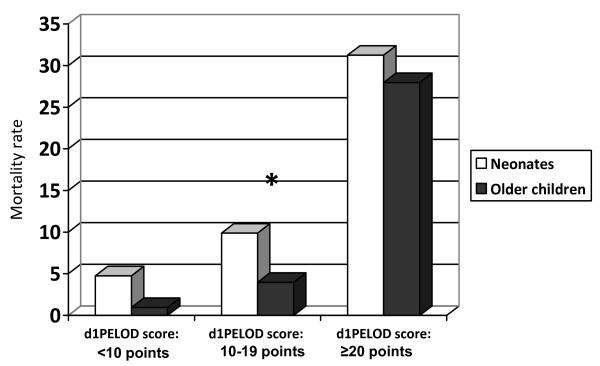
**Pediatric logistic organ dysfunction (PELOD) score values on the first day (d_1_PELOD score) and mortality rate in neonates and older children**. *P < 0.04.

**Figure 2:**
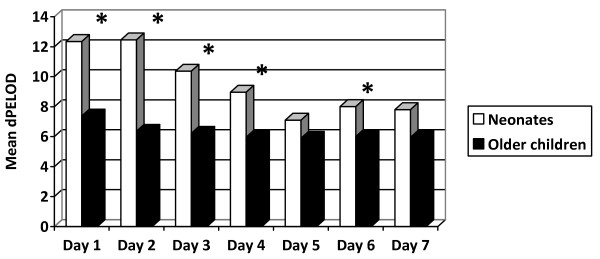
**Mean daily pediatric logistic organ dysfunction (PELOD) score values (mean dPELOD) in neonates and older children**. *Significant difference between the two strata.

The median PELOD score was higher in neonates than older children (5 versus 3, *P *< 10^−4^). The discriminative capacity of the PELOD score was acceptable in neonates (AUC = 0.78) but was excellent among older children (AUC = 0.93); the difference was significant (*P *= 0.008). Within each strata, the mortality rate increased from one class to the other of d_1_PELOD score values (Figure 1). A significant difference in mortality rate between the two strata was found in the medium d_1_PELOD class only (Figure 1). The mean dPELOD scores were higher in neonates from days 1 to 4 (Figure 2). Discriminative values of the dPELOD score from days 1 to 7 were acceptable to excellent in neonates (AUCs = 0.73 to 0.94) but were good among older children (AUCs = 0.84 to 0.89).

### Organ dysfunctions and daily organ dysfunctions

**Figure 3:**
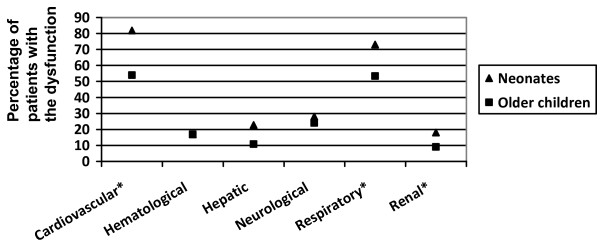
**Incidence of organ dysfunctions during the pediatric intensive care unit stay in neonates and older children**. *Significant differences between the two strata.

**Figure 4:**
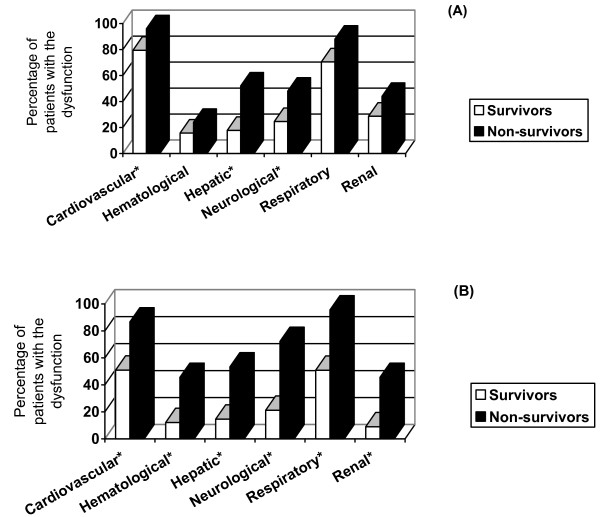
**Frequencies of organ dysfunctions during the pediatric intensive care unit stay among survivors and non-survivors: neonates (a) and older children (b)**. *Significant difference between survivors and non-survivors.

Over the entire PICU stay, neonates presented cardiovascular, respiratory, or renal dysfunctions more frequently (Figure 3). Moreover, cardiovascular, hepatic, and neurological dysfunctions developed more frequently among neonates who did not survive (Figure 4a), whereas all ODs were significantly more frequent among older children who died (Figure 4b).

**Figure 5:**
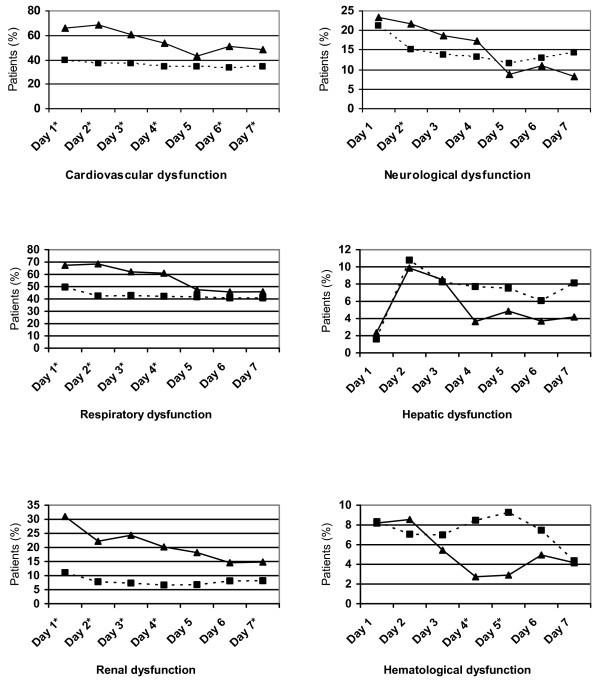
**Incidence of daily organ dysfunctions in neonates (black triangles) and older children (black squares)**. *Significant difference between the two strata.

From days 1 to 4, cardiovascular, respiratory, and renal dysfunctions were significantly more frequent in neonates than older children (Figure 5). In neonates, only cardiovascular, neurological, and hepatic dysfunctions were statistically related to mortality. In older children, all ODs were statistically related to mortality (Table 2). Neurological and cardiovascular dysfunctions accounted for 46% and 28% of the PELOD score variance in neonates and 34% and 47% in older children (Table 2).

**Table 2:** Relative statistical contribution to mortality of each organ dysfunction (logistic regression) and of the PELOD score (multiple regression) in neonates and older children

	Logistic regression, odds ratio (95% CI)				Multiple regression, partial *r^2^*	
**Dysfunction**	**Neonates**		**Older children**		**Neonates**	**Older children**

Neurological	1.118	(1.052-1.188)	1.156	(1.124-1.190)	0.46	0.34
Cardiovascular	1.211	(1.040-1.411)	1.116	(1.048-1.189)	0.28	0.47
Renal	0.970	(0.867-1.086)	1.099	(1.034-1.168)	0.19	0.12
Respiratory	1.126	(0.971-1.307)	1.172	(1.096-1.253)	0.06	0.05
Hematological	1.604	(0.956-2.690)	1.156	(1.019-1.312)	0.01	0.02
Hepatic	3.020	(1.012-9.011)	2.003	(1.115-3.599)	0.001	0.001

CI, confidence interval; PELOD, pediatric logistic organ dysfunction.

## Discussion

This study showed that the incidence rate and severity of MODS and mortality rate were significantly higher in neonates compared with older children. The median and mean dPELOD scores were significantly higher in neonates from days 1 to 4 after admission to the PICU. Cardiovascular, respiratory, and renal dysfunctions were also significantly more frequent in neonates from days 1 to 4. Only three ODs were statistically related to mortality in neonates, whereas all ODs contributed significantly to mortality among older children.

There are a few studies on the incidence of MODS in the PICU. Proulx and colleagues [19] reported an incidence rate of 18%, but no distinction was made between neonates and older children. In the study by Typpo and colleagues [16] (*n *= 44,693 patients; neonates excluded), the incidence of MODS on day 1 was 18.6%, and all ODs contributed to mortality. We found that MODS was more frequent in neonates; this suggests that a stratification for neonates versus older children might be useful in clinical trials in which MODS is considered an outcome measure.

Mortality rate of neonates in the present study, in which prematures were excluded, was higher (14.6%) than mortality rate in neonates of all birth weights, admitted to Canadian neonatal intensive care units (4%) [20]. In our study, the significant mortality difference found between neonates and older children could be attributed to the higher incidence of MODS and a higher PRISM score among neonates compared with older children. This may reflect different diseases leading to PICU admission among neonates compared with older children (Table 1). This difference in mortality might also be attributed to different physiological processes among neonates [21] and the high frequency and variety of congenital anomalies [22].

Even though physiology does not change abruptly, studies have shown differences in organ response to injury between neonates and adults [14,15]. In neonates with MODS, there is an early and prominent microvascular failure, characterized by a generalized capillary leak and anasarca, followed by renal and hepatic dysfunctions, while pulmonary dysfunction is the first to develop in human and animal adult MODS [14,15].

In our study, cardiovascular dysfunction significantly contributed to neonatal mortality. In neonates, cardiomyocyte differs from that in adults because of structural differences, functional alterations in proliferative activity, and excitation-contraction coupling [23]. Cardiac physiology is also quite different: the capacity to increase stroke volume is lower in neonates. These physiological abnormalities, coupled with the fact that the neonatal left ventricular myocardium already functions at a higher baseline contractile state, and the high dependence of left ventricle systolic performance on afterload increase the susceptibility of neonates to sudden cardiac deterioration in the setting of shock and vasoconstriction [23]. Severe congenital cardiac diseases might also explain why the hazard ratio of death attributable to cardiac dysfunction is so high in neonates.

Respiratory dysfunction did not significantly contribute to mortality in neonates but was significantly more frequent in neonates during the first 4 days only. This high frequency of respiratory dysfunction may explain the higher percentage of ventilated neonates compared with that of older children. The contribution of respiratory dysfunction to mortality could have been diminished by recent management development such as extracorporeal membrane oxygenation in newborns [24].

Renal dysfunction was significantly more frequent in neonates during the first 4 days but did not significantly contribute to mortality. This might be explained by the good efficacy of supportive treatment in most cases of neonatal acute renal failure [25]. Factors associated with neonatal mortality in case of renal dysfunction include multiorgan failure, hypotension, need for vasopressors, hemodynamic instability, and need for mechanical ventilation and dialysis [26]. This probably means that death in neonates with renal failure is seldom caused primarily by renal diseases [27].

Hepatic dysfunction significantly contributed to mortality in the two strata (odds ratios [ORs] 3.02, 95% confidence interval [CI] 1.01 to 9.1 in neonates and 2.00, 95% CI 1.12 to 3.60 in older children). However, hepatic dysfunction in neonates and older children had a relatively low incidence (22.8% and 16.8%, respectively). In the study by Tantaleán and colleagues [1] carried out on 276 patients (including 37 newborns) admitted to the PICU, hepatic dysfunction was infrequent (5.8%) and associated with the highest risk of mortality (OR 7.33, 95% CI 1.99 to 26.9) [1]. In the study by Typpo and colleagues [16], hepatic dysfunction had the lowest incidence (0.9%) and the OR of mortality (3.7, 95% CI 2.7 to 5.1) was close to that of the other ODs (from 2.8 [95% CI 2.5 to 3.2] for cardiovascular dysfunction to 5.5 [95% CI 4.7 to 6.5] for respiratory dysfunction).

Neurological dysfunction significantly contributed to mortality in neonates and older children. In the study by Flori and colleagues [28], which included children and neonates who were more than 36 weeks of gestational age and meeting the 1994 American European Consensus Committee definition of acute lung injury (*n *= 328 admissions), neurological dysfunction contributed independently to an increased risk of death (OR 12.58, 95% CI 6.78 to 23.31). In our study, the ORs of 1.118 in neonates and 1.156 in older children corresponded to a variation of 1 point for the neurological OD score. In patients with severe neurological dysfunction (corresponding to 20 points), ORs were 9.31 in neonates (1.118^20^) and 18.16 in older children (1.156^20^). These values were close to the OR of 12.58 reported by Flori and colleagues [28].

Incidence of hematological dysfunction of the entire stay was the same in neonates and older children (17%). Hematological dysfunction was a significant contributor to mortality in older children only (Table 2). Similarly, in children excluding neonates, Johnston and colleagues [29] also showed that hematological dysfunction was a significant contributor to mortality (OR 3.10, 95% CI 2.78 to 3.46).

A limitation of this prospective study is the time elapsed since the period when data were collected (1998, 2000): there is a risk that the case mix of patients in the PICU has changed over this period. A second limitation is the possibility of a false association between the PELOD score and death rate in neonates. In fact, the AUC in this group was acceptable but lower (0.78) than in the older children group (0.93), suggesting a differential performance (discrimination) of the model between the two groups. Another limitation is that we considered only intensive care unit mortality and not hospital mortality. However, in hospital post-intensive care, mortality of critically ill children is not frequent. A study from a PICU, typical of US units, showed that among 341 survivors only three children (0.9%) died in the hospital after discharge from intensive care [30]. Otherwise, the PELOD score has been criticized, mainly because it does not assign risk on a continuous scale [31].

## Conclusions

The incidence and severity of MODS and mortality rate were significantly higher in neonates than in older children. Three ODs - neurological, cardiovascular, and hepatic dysfunctions - significantly contributed to mortality in neonates, whereas all ODs were significantly associated with mortality in older children. In our hands, the PELOD and dPELOD scores were higher in neonates and risk of death with similar PELOD scores tended to be higher in neonates than older children. These data suggest that an updated version of the PELOD score should take this into account; also, they suggest that it might be a good strategy to consider these two strata in randomized clinical trials involving critically ill children.

## Key messages

• In the pediatric intensive care unit, mortality is higher among neonates (excluding prematures) compared with older children.

• Incidence of multiple organ dysfunction syndrome is higher among neonates (excluding prematures) than in older children.

• In neonates, neurological, cardiovascular, and hepatic dysfunctions are the main predictors of death, whereas all organ dysfunctions contribute to mortality in older children.

• Stratification for neonates versus older children might be useful in clinical trials in which organ dysfunction score is the outcome measure.

## Abbreviations

AUC: area under the receiver operator characteristic curve; CI: confidence interval; dOD: daily organ dysfunction; dPELOD: daily pediatric logistic organ dysfunction; MODS: multiple organ dysfunction syndrome; OD: organ dysfunction; OR: odds ratio; PELOD: pediatric logistic organ dysfunction; PICU: pediatric intensive care unit; PRISM: pediatric risk of mortality.

## Competing interests

The authors declare that they have no competing interests.

## Authors' contributions

SL contributed to study conception and design, to the acquisition, analysis, and interpretation of data, and to drafting the article and provided statistical expertise. FL contributed to study conception and design, to analysis and interpretation of data, and to drafting the article and obtained funding and provided supervision. BG contributed to study conception and design and to analysis and interpretation of data and provided statistical expertise. FP contributed to the acquisition of data. NB and JL contributed to analysis and interpretation of data and to drafting the article. AD contributed to analysis and interpretation of data and to drafting the article and provided statistical expertise. All investigators commented on, critically revised, and read and approved the final manuscript.
